# miR-539 inhibits prostate cancer progression by directly targeting SPAG5

**DOI:** 10.1186/s13046-016-0337-8

**Published:** 2016-04-01

**Authors:** Hongtuan Zhang, Shadan Li, Xiong Yang, Baomin Qiao, Zhihong Zhang, Yong Xu

**Affiliations:** Department of Urology, National Key Specialty of Urology, Second Hospital of Tianjin Medical University, Tianjin Key Institute of Urology, Tianjin Medical University, Tianjin, China; Vancouver Prostate Centre & Department of Urologic Sciences, Faculty of Medicine, University of British Columbia, Vancouver, BC Canada; Department of Urology, Chengdu military general hospital, Chendu, Sichuan China

**Keywords:** miRNA, Progression, miR-539, Prostate cancer, SPAG5

## Abstract

**Background:**

We conducted multiple microarray datasets analyses from clinical and xenograft tumor tissues to search for disease progression-driving oncogenes in prostate cancer (PCa). Sperm-associated antigen 5 (SPAG5) attracted our attention. SPAG5 was recently identified as an oncogene participating in lung cancer and cervical cancer progression. However, the roles of SPAG5 in PCa progression remain unknown.

**Methods:**

SPAG5 expression level in clinical primary PCa, metastatic PCa, castration resistant PCa, neuroendocrine PCa, and normal prostate tissues was investigated. We established multiple in vivo xenografts models using patient-derived tissues and investigated SPAG5 expression trend in these models. We also investigated the functions of SPAG5 in vivo and in vitro studies. Luciferase reporter assays were performed to investigate potential miRNAs that can regulate SPAG5.

**Results:**

We identified that SPAG5 expression was gradually increased in PCa progression and its level was significantly associated with lymph node metastasis, clinical stage, Gleason score, and biochemical recurrence. Our results indicated that SPAG5 knockdown can drastically inhibit PCa cell proliferation, migration, and invasion in vitro and supress tumor growth and metastasis in vivo. We identified that miR-539 can directly target SPAG5. Ectopic overexpression of miR-539 can drastically inhibit SPAG5 expression and the restoration of SPAG5 expression can reverse the inhibitory effects of miR-539 on PCa cell proliferation and metastasis.

**Conclusion:**

Our results collectively showed a progression-driving role of SPAG5 in PCa which can be regulated by miR-539, suggesting that miR-539/SPAG5 can serve as a potential therapeutic target for PCa.

**Electronic supplementary material:**

The online version of this article (doi:10.1186/s13046-016-0337-8) contains supplementary material, which is available to authorized users.

## Background

Prostate cancer (PCa) is one of the most common and aggressive human malignancies with poor prognosis worldwide [1]. Although considerable advances in therapy and diagnosis of PCa have been made, many PCa patients will progress to castration-resistant PCa (CRPC) that is metastatic and shows resistance to hormonal therapy [2]. Neuroendocrine PCa (NEPC) is another lethal form of PCa with most patients dying within 1 year of diagnosis despite very aggressive chemotherapeutic regimens [3]. Increasing evidence indicates that prostatic adenocarcinoma can undergo a NE transdifferentiation following long-term androgen deprivation therapy, and eventually progress to NEPC [3, 4]. However, NEPC studies have been hampered by a lack of clinically relevant in vivo models of the disease. Recently, our lab successfully has generated a patient-derived xenograft model of complete neuroendocrine transdifferentiation (LTL331R) of a prostate adenocarcinoma (LTL331) at the Living Tumor Laboratory [5]. Therefore, NEPC xenograft model provides a valuable method for investigating the molecular mechanisms of NEPC progression. So discovery of new optimize therapeutic targets for more effective treatment of metastatic PCa, CRPC, and NEPC is urgently needed for improved disease management and patient survival [6].

It is well known that sperm-associated antigen 5 (SPAG5) can bind to microtubules as a regulator of the timing of spindle organization and separation of sister chromatids [7]. Previous studies indicated that SPAG5 was an important variable in mitosis and cell cycle checkpoint regulation [8]. A previous study indicated that SPAG5 may act as a promoter in tumorigenesis and progression [9]. Previous studies showed that SPAG5 overexpression can predict poor prognosis in lung cancer and cervical cancer [9–11] and alter sensitivity to taxol treatment via the mTOR signaling pathway in cervical cancer [11]. These results indicated that SPAG5 may act as an important oncogene that is involved in the formation and progression of malignancies and might influence the biological behaviors of malignancies. However, the molecular mechanism and role of SPAG5 in PCa development and progression have not yet been investigated.

miRNAs are small noncoding RNAs of ~22 nt that can regulate gene expression by inhibiting protein translation and promoting the degradation of the target mRNAs by binding to the 3’ untranslated regions (UTR) of specific mRNAs. An expanding body of evidence has showed that the dysregulation of miRNAs is linked to the development of various types of cancers [12–16]. Increasing evidence indicates a critical role for miRNAs in cancer initiation, promotion, and progression [17–19]. In view of the extensive functions of miRNAs and SPAG5, there is an urgent need to identify the molecular mechanism and role of SPAG5 in PCa progression and to investigate whether or not SPAG5 can be targeted by a specific miRNA in PCa.

In the current study, we identified that SPAG5 expression level is drastically increased in primary PCa relative to normal samples, metastatic PCa samples relative to primary PCa, CRPC relative to hormone naïve PCa, and NEPC relative to prostate adenocarcinoma, respectively. We also identified that knockdown of SPAG5 significantly inhibited the proliferation, migration, and invasion in PCa cells. We further confirmed that miR-539 inhibited the PCa growth and metastasis in vivo and also inhibited the proliferation, migration, and invasion in vitro by down-regulating SPAG5 expression. Taken together, our research results position SPAG5 as a progression-driving oncogene in PCa and a potential therapeutic target in PCa, providing opportunities to explore novel strategies aimed at reversing PCa progression.

## Methods

### Tissue samples

One hundred eighty PCa and paired adjacent normal tissues confirmed by pathologists were collected from the second hospital of Tianjin medical university [20, 21]. We also collected 20 CRPC tissues. Informed consent was obtained from each patient, and all of the experiments were approved by the ethics committee of the institute.

### PCa cell lines

PC-3 (a cell line characteristic of prostatic small cell neuroendocrine carcinoma) and LNCaP cell lines were cultured in RPMI 1640 (Life Technologies, CA) with 0.023 IU/ml insulin and 10 % FBS (Invitrogen) in 5 % CO_2_ cell culture incubator.

### Plasmid construction

We amplified a DNA fragment containing the pri-miR-539 gene DNA from genomic human DNA by PCR. This fragment was then inserted into pcDNA3 vector. The primers for knocking down SPAG5 were synthesized, annealed, and cloned into the pSilencer2.1-neo vector. The SPAG5 cDNA containing the coding sequence was cloned by PCR, and the PCR product was cloned into the pcDNA3 vector.

### Colony formation assay

We seed the PCa cells on 35-mm dishes according to the manufacturers’ instructions. The PCa cells were fixed in methanol, and then stained with crystal according to the manufacturers’ instructions. Positive colony formation (>50 cells/colony) would be counted.

### Migration and invasion assays

The transwell migration and invasion assays were conducted with coated Matrigel (invasion) and uncoated (migration) according to the manufacturers’ instructions. The migrated and invaded cells in the membrane would be fixed, stained, and counted.

### Luciferase reporter assay

The miR-539-binding site in the SPAG5 3’-UTR region (wild or mutant-type) was cloned downstream of the firefly luciferase gene in a pGL3-promoter vector. The luciferase assay was conducted according to the manufacturers’ instructions.

### qRT-PCR

Total RNA was extracted using Trizol Reagent according to the manufacturers’ instructions. The miR-539 expression was measured by TaqMan miRNA assays according to the manufacturers’ instructions, U6 was used for normalization. SPAG5 mRNA expression level was investigated by SYBR green qPCR assay and β-actin was used as an endogenous control.

### Western blots

Detailed procedure for western blot was performed according to the manufacturers’ instructions. In brief, the interest protein was resolved by SDS-PAGE and transferred to a PVDF membrane, which was probed with specific primary antibody against SPAG5 (HPA022479, Sigma). β-actin was used as the endogenous control.

### Immunohistochemistry

The Immunohistochemistry study was conducted according to the manufacturers’ instructions using specific primary antibody against SPAG5 (HPA022479, Sigma). The SPAG5 protein would be classified semiquantitatively combining the proportion and intensity of positively stained immunoreactive cells [20, 21]. The sum of the staining intensity score and the percentage score was used to define SPAG5 protein level: 0–2, low expression and 3–4, high expression [20, 21].

### PCa xenograft research

The LNCaP and PC-3 cells were stably transfected with pri-miR-539, control, or SPAG5 knockdown vector. The cells were subcutaneously injected into the 6 week-old nude mice in the flank. Mouse weight and tumor size were measured biweekly. The tumor volume was calculated as follows: length × width^2^ × 1/2. The xenograft tumors and cervical lymph nodes samples were collected and tumor weights were measured 7 weeks after implantation. DNA extraction of the cervical lymph nodes and human alu sequence PCR amplification were performed to identify the distant metastasis status according to a previous paper [22].

### Statistical analysis

Student’s *t*-test was conducted for continuous data. Spearman correlation test was used to investigate the associations between SPAG5 protein level and the clinical and pathological factors. Survival curves were conducted by the Kaplan-Meier and investigated through log-rank test. Identified variables were associated with survival by the Cox proportional hazard regression. A p value less than 0.05 was considered significant. Statistical analysis was performed by SPSS 17.0.

## Results

### SPAG5 may be a potential progression-driving oncogene

**Fig. 1:**
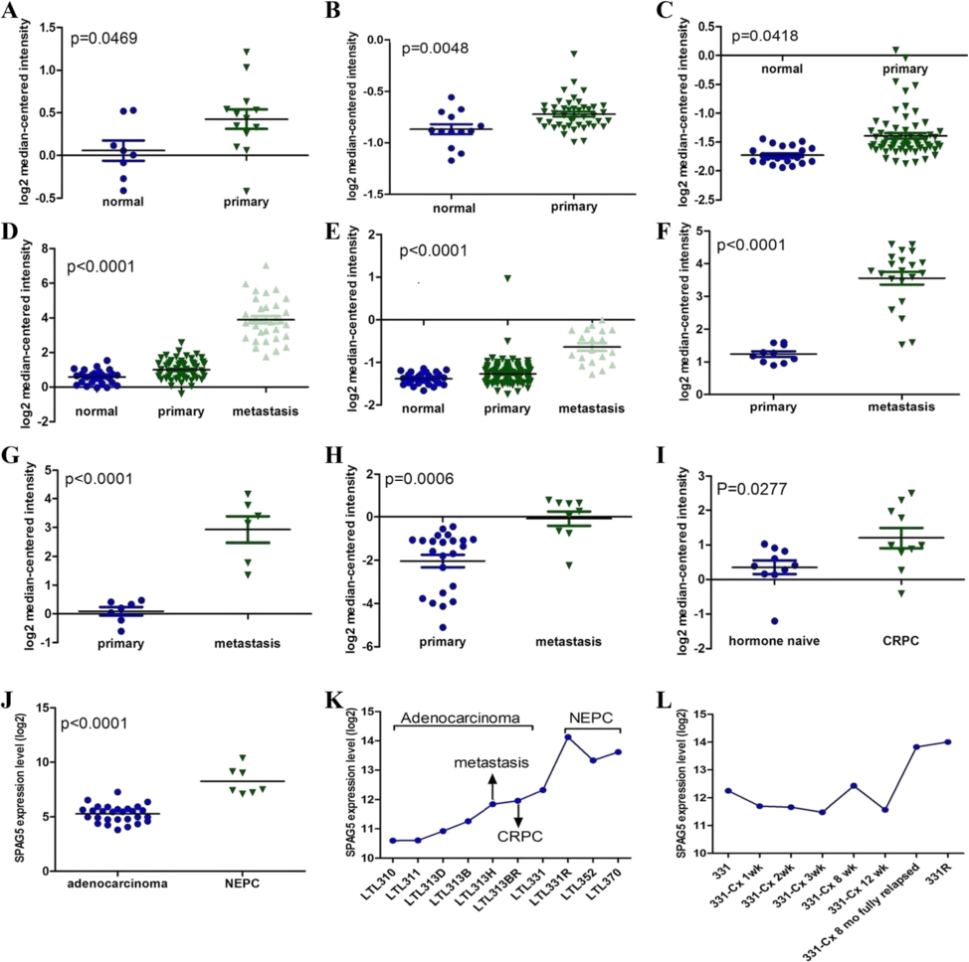
SPAG5 is gradually increased in normal prostate, primary PCa, metastatic PCa, CRPC, and NEPC. **a**-**e**, Scatter plots represent SPAG5 mRNA in normal prostate and primary PCa samples; **d**-**h**, and **i**, Scatter plots represent SPAG5 mRNA in primary PCa and metastatic PCa samples; **i**, Scatter plots represent SPAG5 mRNA in metastatic PCa and CRPC samples; **j.** Scatter plots represent SPAG5 mRNA in prostate adernocarcinoma and NEPC samples; **k**, Scatter plots showed that SPAG5 mRNA was upregulated in three NEPC models (LTL331R, LTL352, and LTL370) relative to these prostate adenocarcinoma models, metastatic (LTL313H) PCa xenograft line relative to non-metastatic (LTL313B) PCa xenograft line, CRPC xenograft model (313BR) compared with LTL313B; **l**, Scatter plots showed that SPAG5 was downregulated after host castration and significantly upregulated in fully relapsed NEPC xenografts

In order to investigate whether any difference of SPAG5 expression exists in primary PCa, metastatic PCa, CRPC, NEPC, and normal prostate tissues, several microarray datasets were analyzed [23–30]. We identified that SPAG5 mRNA was significantly overexpressed in primary PCa relative to normal prostate tissues (Fig. 1a-e) and metastatic PCa compared with primary PCa samples, respectively (Fig. 1d-h). The microarray data also showed that SPAG5 mRNA was higher in CRPC samples than in hormone naïve PCa samples (Fig. 1i). We also investigated SPAG5 expression in a clinical cohort that included 30 prostate adenocarcinoma and 7 NEPC patients. The RNA-seq data of this cohort showed that SPAG5 was significantly upregulated in NEPC samples compared with prostate adenocarcinoma samples (Fig. 1j). In order to validate clinical cohort results, we further investigate SPAG5 mRNA expression in a panel of patient tissue-derived PCa xenograft models generated from our lab. The results indicated that SPAG5 mRNA was upregulated in three NEPC models (LTL331R, LTL352, and LTL370) relative to these prostate adenocarcinoma models (Fig. 1k). We also analyzed gene expression profile of paired metastatic (LTL313H) and non-metastatic (LTL313B) PCa tissue xenograft lines derived from one patient’s primary tumor, LTL313H showed higher expression of SPAG5 relative to LTL313B. Similarly, increased SPAG5 mRNA was identified in CRPC xenograft model (313BR) compared with LTL313B (Fig. 1k). Our lab also has analysed the microarray data from LTL331 xenografts before and after host castration at various time points. We identified that SPAG5 was downregulated after host castration and significantly upregulated in fully relapsed NEPC xenografts (Fig. 1l). Collectively, above data indicated that SPAG5 increases gradually during the PCa progression, indicating it has a very critical role in PCa progression.

### SPAG5 is significantly associated with PCa progression and poor prognosis

**Fig. 2:**
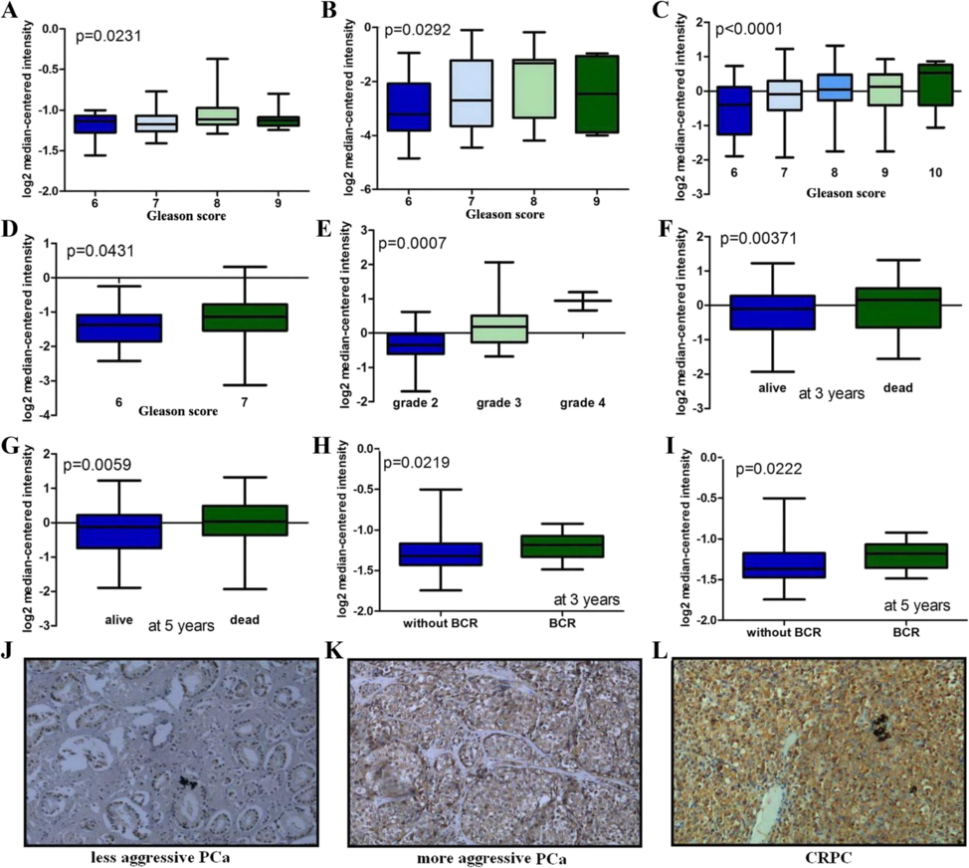
SPAG5 overexpression is associated with PCa progression and poor prognosis. **a**-**d**, Box plots showed that SPAG5 was significantly increased in patients with higher Gleason score relative to patients with lower Gleason score; **e**, Box plots showed that SPAG5 was significantly increased in patients with higher grade relative to patients with lower grade; **f, g**, Box plots showed that SPAG5 mRNA was significantly overexpressed in dead PCa patients compared with living PCa patients at 3 years and 5 years, respectively; **h, i**, Box plots showed that SPAG5 mRNA was significantly increased in PCa patients with BCR compared with PCa patients without BCR at 3 years, and 5 years, respectively. **j**-**l**, Immunohistochemical analysis of SPAG5 in, less aggressive PCa (**j**), aggressive PCa (**h**), and CRPC (**l**)

We also performed microarray analysis to identify whether SPAG5 was associated with PCa progression [30–33]. We identified that high SPAG5 expression was significantly associated with higher PCa grade, and higher stage (Fig. 2a-e). SPAG5 expression was significantly overexpressed in dead PCa patients compared with living PCa patients at 3 years and 5 years, respectively (Fig. 2f, g). We also identified that SPAG5 expression was significantly increased in PCa patients with BCR compared with PCa patients without BCR at 5 years and 3 years, respectively (Fig. 2h, i). In order to validate these results, we further conducted immunohistochemistry study to investigate SPAG5 protein expression in PCa. SPAG5 protein expression was increased in PCa tissues compared with the benign prostate hyperplasia, and the difference was significant. As shown in Fig. 2j-k, the SPAG5 positive staining was localized within the cytoplasm. The analysis indicated weak or no reactivity in less aggressive PCa but strong staining in the aggressive PCa and CRPC samples.

We investigated the association between SPAG5 protein expression and clinicopathological variables. As shown in Additional file 1: Table S1, high SPAG5 expression was significantly associated with lymph node metastasis, clinical stage, Gleason score, and BCR. We further identified that the high SPAG5 expression patients had a shorter overall or BCR-free survival duration compared to the patients with low SPAG5 expression. We also identified that SPAG5 was an independent prognostic variable for BCR-free and overall survival of PCa patients (Additional file 2: Table S2 and Additional file 3: Table S3). Collectively, our data showed high SPAG5 protein expression is significantly associated with PCa progression and poor prognosis.

### SPAG5 silence inhibits PCa cell colony formation, migration, and invasion

To investigate the functional significance of SPAG5 overexpression in PCa, we reduced SPAG5 expression in PCa cells and studied its impacts on cell colony formation, migration, and invasion. We employed stable knockdown strategy targeting SPAG5 in PCa cells. We identified significant decrease in colony formation in SPAG5 silence cells relative to control cells (Additional file 4: Figure S1A). We also identified that SPAG5 silence significantly inhibited PC-3 and LNCaP cell migration and invasion (Additional file 4: Figure S1B, C). We confirmed that SPAG5 protein was significantly reduced in SPAG5 silence cells compared with control cells (Additional file 4: Figure S1D).

### SPAG5 is a direct target of miR-539 which may be a potential anti-progression miRNA

**Fig. 3:**
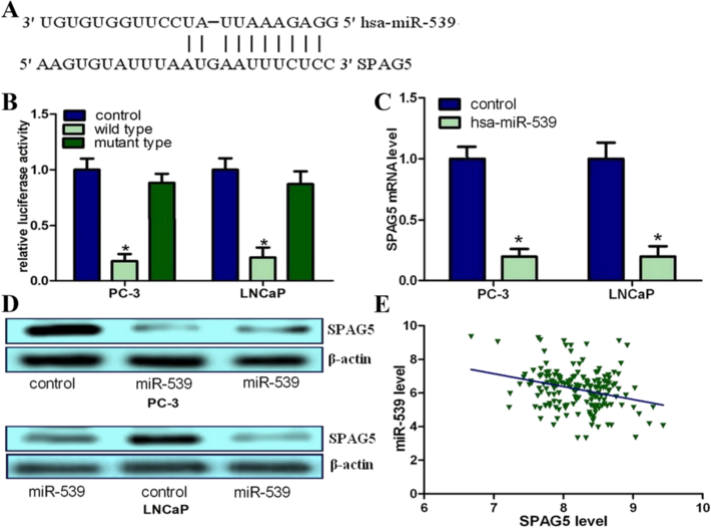
SPAG5 is a direct target of miR-539 in PCa. **a**, Computational analysis indicating that miR-539 potentially targeted SPAG5. **b**, Relative luciferase activities were studied in PCa cells. **c**, Decrease in SPAG5 mRNA expression by miR-539 was investigated using qRT-PCR. **d**, Decrease in SPAG5 protein expression by miR-539 was investigated using western blots. **e**, SPAG5 mRNA was inversely associated with miR-539 in 180 pairs of PCa tissues

We employed four miRNA target prediction algorithms provided by miRanda, miRWalk, PICTAR5, and Targetscan. After integrating the results, we select miR-539 for further research because of its tumor suppressor properties in osteosarcoma and thyroid cancer [34, 35]. However, up to now, its roles in PCa and other cancers are still unclear. We identified that miR-539 is significantly decreased in both primary PCa compared with normal prostate tissues and metastatic PCa relative to primary PCa tissues (Additional file 5: Figure S2) [30]. The binding site for miR-539 at 3’-UTR of SPAG5 was depicted (Fig. 3a). We found that co-transfection with miR-539 can inhibit the luciferase activity when the construct contained the 3’UTR of SPAG5 (Fig. 3b). Mutation of the binding site can reverse the effects. Taken together, above results indicated that SPAG5 was a direct target of miR-539. We further performed qRT-PCR and western blot to study whether ectopic overexpression of miR-539 can change SPAG5 expression. We identified that SPAG5 mRNA expression was significantly downregulated in miR-539 transfectants as compared with control cells (Fig. 3c). We also identified that SPAG5 mRNA expression was inversely associated with the miR-539 expression level (Fig. 3d). Overall, these results showed that miR-539 is gradually decreased in PCa progression and may directly target SPAG5.

### miR-539 inhibits PCa cell proliferation, invasion, and migration by directly targeting SPAG5

We investigated whether miR-539 can change the effects of SPAG5 on PCa cell colony formation, invasion, and migration. We found that SPAG5 overexpression can significantly abrogate the inhibitory effects of PCa cell colony formation induced by miR-539 (Additional file 4: Figure S1A). We also found that SPAG5 overexpression can reverse the inhibitory effects of PCa cell migration and invasion induced by miR-539 (Additional file 4: Figure S1B, C). The efficiency of miR-539 upregulation was studied by western blots (Additional file 4: Figure S1D). We found that SPAG5 knockdown can result in similar effects induced by miR-539 upregulation in PCa cells (Additional file 4: Figure S1).

### SPAG5 plays a critical role in PCa growth and metastasis in vivo

**Fig. 4:**
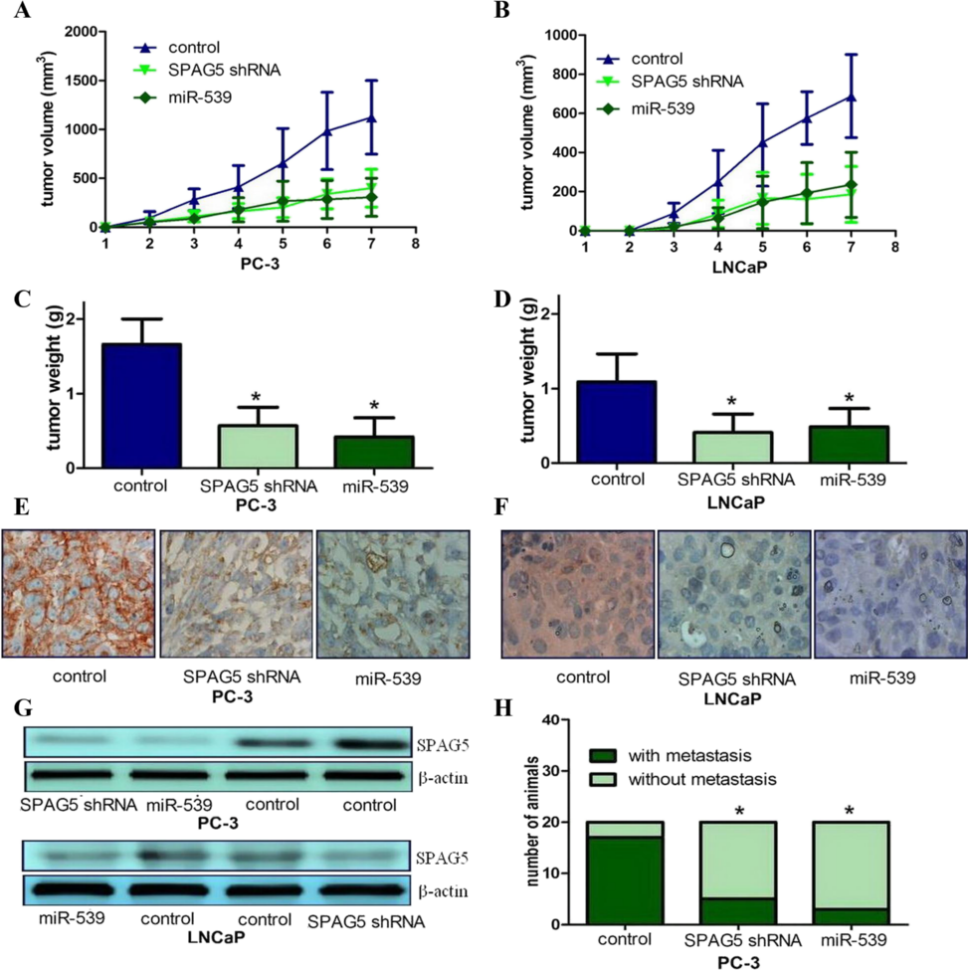
SPAG5 plays a critical role in PCa growth and metastasis in vivo. **a, b**, SPAG5 knockdown in PCa cells significantly inhibits tumor growth in a mouse xenograft model; **c, d**, Tumor weights of corresponding mouse xenograft models; **e**-**g**, SPAG5 expression analysis was conducted at protein level by immunohistochemical analysis and western blot. **h**, SPAG5 played an important role in PCa metastasis in vivo. Ectopic expression of miR-539 can mimic the effects induced by SPAG5 knockdown. All * indicated *P* value less than 0.05

To study the role of SPAG5 on PCa growth and metastasis in vivo, a murine xenograft model using stable SPAG5 silence PCa cells was employed. SPAG5 silence can lead to significantly reduced tumor volume and weight relative to control mice (Fig. 4a-d). SPAG5 silence tumor tissues showed reduced staining for SPAG5 (Fig. 4e-g). SPAG5 silence significantly inhibited PC-3 cell’s ability to metastasize relative to control group (Fig. 4h). miR-539 upregulation can result in attenuated metastasis relative to the control group, and lead to similar results caused by SPAG5 knockdown (Fig. 4). We did not find any metastases in control or experimental LNCaP xenografts. Collectively, above data suggested that SPAG5 may play a critical role in PCa growth and metastasis in vivo.

## Discussion

It is well known that surgery and radiation therapy can cure the localized PCa [36, 37]. However, many treated PCa patients will experience local recurrence or metastasis [2–4]. Metastatic PCa, CRPC, and NEPC are highly resistant to conventional therapy and are at present incurable [36, 38, 39]. We know that most NEPC patients result from transdifferentiation of a typical prostate adenocarcinoma following androgen-deprivation therapy. We have recently established the first in vivo model of complete neuroendocrine transdifferentiation using patient-derived xenografts. Development of therapeutic approaches specifically targeting metastatic PCa, CRPC, and NEPC associated genes may lead to improved PCa management. Previous study reported overexpression of SPAG5 in several types of cancers [9–11]. However, there has been no report about SPAG5 functional analyses in PCa. In current study, we try our best to investigate SPAG5 expression pattern and its association with PCa carcinogenesis, progression, and prognosis. Our data strongly indicates that SPAG5 functions as a progression-driving oncogene in PCa.

We first found a significant upregulation of SPAG5 expression in primary PCa relative to normal prostate tissues, metastatic PCa relative to primary PCa, CRPC relative to hormone naïve PCa, and NEPC relative to prostate adenocarcinoma through microarray analysis, indicating SPAG5 may has a critical role in PCa progression. In order to find the potential critical role of SPAG5 in PCa, we further studied the association of SPAG5 protein staining with clinicopathological variables in PCa. Results showed that SPAG5 positive staining was significantly associated with PCa progression. Results also suggested that positive staining of SPAG5 was independently associated with unfavorable outcome of PCa patients. The prognostic value of SPAG5 was statistically significant in multivariate analysis adjusted for significant variables from univariate analysis, which suggested SPAG5 expression may be a good molecular marker to predict PCa prognosis. This is the first direct evidence of the association between SPAG5 and clinicopathological variables of PCa and the prognostic role of SPAG5 in PCa.

In support of our observations in clinical research, we identified important functional roles of SPAG5 in PCa cells. Besides the reduction of PCa cell colony formation, we also found impaired migration and invasion abilities of PC-3 and LNCaP cells upon inducible shRNA-mediated SPAG5 silence, indicating that the SPAG5 functioned as a progression associated gene in PCa. We further confirmed that SPAG5 knockdown can significantly inhibit tumor growth and metastasis in vivo. Increasing evidence showed miRNAs have critical roles in several biological processes, including differentiation, progression, angiogenesis, proliferation, migration, and invasion. We employed 4 public miRNA target prediction algorithms to identify potential miRNAs which can regulate SPAG5. Among of these potential miRNAs, miR-539 attracted our attention due to its tumor suppressor role in osteosarcoma and thyroid cancer [34], and its unclear roles in PCa and other cancers. The data from the Taylor et al. study confirmed that miR-539 expression is low in the more advanced primary PCa, with the lowest expression in metastatic tissues. We further identified that miR-539 level was inversely associated with SPAG5 mRNA level. Our results further confirmed that the miR-539 can directly target SPAG5. As expectedly, ectopic expression of miR-539 can result in similar results caused by SPAG5 silence in PCa cells. Furthermore, ectopic overexpression of SPAG5 can significantly reverse the inhibitory effects of miR-539. Taken together, above results indicated that miR-539 can supress PCa cell colony formation, migration, and invasion by directly targeting SPAG5.

We further investigated the role of SPAG5 on PCa growth and metastasis in vivo using a murine xenograft model. We found that SPAG5 knockdown can contribute to decreased xenograft tumor volume and weight relative to control group. We also found that SPAG5 knockdown can inhibit PC-3 cell’s ability to metastasize relative to control group. As expected, miR-539 overexpression can lead to attenuated metastasis relative to the control group, and lead to similar results induced by SPAG5 knockdown. Taken together, above results indicated that SPAG5 may play a critical role in PCa growth and metastasis in vivo.

## Conclusions

To our best knowledge, this is the first study to report the potential progression-driving function of SPAG5 in PCa. Our study also provides first experimental evidence that miR-539 can inhibit PCa cell growth and metastasis in PCa by directly repressing SPAG5. The newly identified miR-539/SPAG5 signal pathway may provide new insights into the progression of PCa and a potential therapeutic target for PCa treatment. Overall, above results indicated that SPAG5 may play a progression-driving role in PCa and could serve as a viable therapeutic target.

## Additional files

Additional file 1: Table S1. Clinicopathologic factors and SPAG5 protein expression in 180 PCa patients. (DOC 68 kb) Additional file 2: Table S2. Prognostic value of SPAG5 protein expression for the biochemical recurrence free survival in univariate and multivariate analyses by Cox regression. (DOC 34 kb) Additional file 3: Table S3. Prognostic value of SPAG5 protein expression for the overall survival in univariate and multivariate analyses by Cox regression. (DOC 34 kb) Additional file 4: Figure S1. miR-539 inhibits PCa cell proliferation, migration and invasion by targeting SPAG5 in vitro. A, Ectopic expression of miR-539 can mimic the suppression of colony formation induced by SPAG5 knockdown in PC-3 and LNCaP cells; B, Ectopic expression of miR-539 can mimic the suppression of migration activity induced by SPAG5 knockdown in PC-3 and LNCaP cells; C, Ectopic expression of miR-539 can mimic the suppression of invasion activity induced by SPAG5 knockdown in PC-3 and LNCaP cells; D, The efficiency of SPAG5 knockdown and ectopic expression of miR-539 was confirmed at protein level by western blot. (JPG 145 kb) Additional file 5: Figure S2. miR-539 is downexpressed in primary PCa and metastatic PCa. miR-539 level was gradually decreased in normal prostate, primary PCa, and metastatic PCa samples. (JPG 173 kb)

## Abbreviations

BCR: biochemical recurrence
CI: confidence interval
CRPC: castration-resistant prostate cancer
NEPC: neuroendocrine prostate cancer
PCa: prostate cancer
PSA: prostate-specific antigen
SPAG5: sperm-associated antigen 5
