# Supplementary material for: miR-539 inhibits prostate cancer progression by directly targeting SPAG5
Source: J Exp Clin Cancer Res. 2016 Apr 1;35:60. doi: 10.1186/s13046-016-0337-8 (PMC4818461; doi:10.1186/s13046-016-0337-8)
Supplement: Additional file 1: Table S1. — Clinicopathologic factors and SPAG5 protein expression in 180 PCa patients. (DOC 68 kb) [file 13046_2016_337_MOESM1_ESM.doc]

**Table S1 Clinicopathologic variables and SPAG5 protein** expression in 180 PCa patients

|  |  | SPAG5 protein | | |  |
| --- | --- | --- | --- | --- | --- |
| Variable | Group | n | High | Low | P value |
| Age |  |  | 58 | 122 | 0.812 |
|  | ＜70 | 97 | 32 | 65 |  |
|  | ≥70 | 83 | 26 | 57 |  |
| Lymph node metastasis |  |  |  |  | <0.001 |
|  | Presence | 17 | 13 | 4 |  |
|  | Absence | 163 | 45 | 118 |  |
| Surgical margin status |  |  |  |  | 0.761 |
|  | Presence | 14 | 4 | 10 |  |
|  | Absence | 166 | 54 | 112 |  |
| Seminal vesicle invasion |  |  |  |  | 0.771 |
|  | Presence | 35 | 12 | 23 |  |
|  | Absence | 145 | 46 | 99 |  |
| Clinical stage |  |  |  |  | <0.001 |
|  | T1 | 103 | 21 | 82 |  |
|  | T2/T3 | 77 | 37 | 40 |  |
| Preoperative PSA |  |  |  |  | 0.364 |
|  | 0-10 | 69 | 25 | 44 |  |
|  | ＞10 | 111 | 33 | 78 |  |
| Gleason score |  |  |  |  | <0.001 |
|  | ＜7 | 99 | 20 | 79 |  |
|  | ≥7 | 81 | 38 | 43 |  |
| Angiolymphatic invasion |  |  |  |  | 0.911 |
|  | Presence | 35 | 11 | 24 |  |
|  | Absence | 145 | 47 | 98 |  |
| Biochemical recurrence |  |  |  |  | <0.001 |
|  | Absence | 128 | 27 | 101 |  |
|  | Presence | 52 | 31 | 21 |  |
