# Supplementary material for: miR-539 inhibits prostate cancer progression by directly targeting SPAG5
Source: J Exp Clin Cancer Res. 2016 Apr 1;35:60. doi: 10.1186/s13046-016-0337-8 (PMC4818461; doi:10.1186/s13046-016-0337-8)
Supplement: Additional file 2: Table S2. — Prognostic value of SPAG5 protein expression for the biochemical recurrence free survival in univariate and multivariate analyses by Cox regression. (DOC 34 kb) [file 13046_2016_337_MOESM2_ESM.doc]

**Table S2 Prognostic value of SPAG5 protein expression for the biochemical recurrence-free survival in univariate and multivariate analyses by Cox regression**

|  | Univariate analysis | | | Multivariate analysis | | |
| --- | --- | --- | --- | --- | --- | --- |
| Covariant | Exp (B) | 95% CI | P value | Exp (B) | 95% CI | P value |
| SPAG5 protein | 3.215 | 1.352-7.6451 | 0.008 | 4.571 | 2.492-8.384 | <0.001 |
| Gleason score | 1.703 | 1.280-2.265 | <0.001 | 2.364 | 1.012-5.522 | 0.047 |
| Preoperative PSA | 1.241 | 0.705-2.188 | 0.454 |  |  |  |
| Age | 1.068 | 0.804-1.419 | 0.650 |  |  |  |
| Angiolymphatic invasion | 1.084 | 0.814-1.443 | 0.580 |  |  |  |
| Surgical margin status | 1.017 | 0.709-1.459 | 0.925 |  |  |  |
| PCa Stage | 1.090 | 0.921-1.291 | 0.316 |  |  |  |
| Lymph node metastasis | 1.140 | 0.850-1.528 | 0.381 |  |  |  |
| Seminal vesicle invasion | 1.505 | 1.132-2.003 | 0.005 | 1.982 | 1.167-3.366 | 0.011 |
