# Supplementary material for: miR-539 inhibits prostate cancer progression by directly targeting SPAG5
Source: J Exp Clin Cancer Res. 2016 Apr 1;35:60. doi: 10.1186/s13046-016-0337-8 (PMC4818461; doi:10.1186/s13046-016-0337-8)
Supplement: Additional file 3: Table S3. — Prognostic value of SPAG5 protein expression for the overall survival in univariate and multivariate analyses by Cox regression. (DOC 34 kb) [file 13046_2016_337_MOESM3_ESM.doc]

**Table S3 Prognostic value of SPAG5 protein expression for the overall survival in univariate and multivariate analyses by Cox regression**

|  | Univariate analysis | | | Multivariate analysis | | |
| --- | --- | --- | --- | --- | --- | --- |
| Covariant | Exp (B) | 95% CI | P value | Exp (B) | 95% CI | P value |
| SPAG5 protein | 4.836 | 2.690-8.694 | <0.001 | 5.106 | 3.123-8.348 | <0.001 |
| Gleason score | 2.526 | 1.788-3.568 | <0.001 | 2.533 | 1.793-3.579 | <0.001 |
| Preoperative PSA | 2.034 | 1.338-23.092 | 0.001 | 1.236 | 0.991-1.542 | 0.060 |
| Age | 1.282 | 0.917-1.792 | 0.146 |  |  |  |
| Angiolymphatic invasion | 1.373 | 0.813-2.319 | 0.235 |  |  |  |
| Surgical margin status | 1.101 | 0.703-1.724 | 0.674 |  |  |  |
| PCa Stage | 4.131 | 2.888-5.911 | <0.001 | 2.984 | 2.162-4.119 | <0.001 |
| Lymph node metastasis | 1.044 | 0.746-1.462 | 0.800 |  |  |  |
| Seminal vesicle invasion | 1.358 | 0.956-1.928 | 0.087 |  |  |  |
