# Supplementary figures and images for: miR-539 inhibits prostate cancer progression by directly targeting SPAG5
Source: J Exp Clin Cancer Res. 2016 Apr 1;35:60. doi: 10.1186/s13046-016-0337-8 (PMC4818461; doi:10.1186/s13046-016-0337-8)

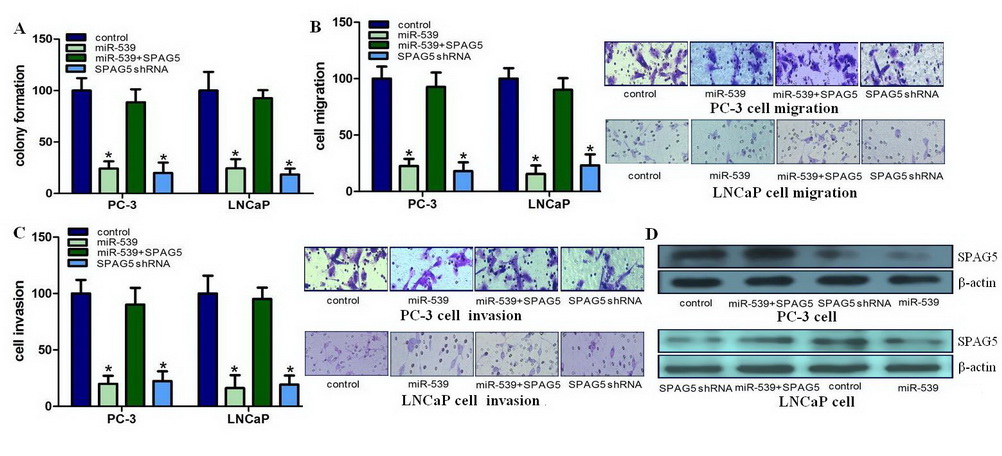

Supplement: Additional file 4: Figure S1. — miR-539 inhibits PCa cell proliferation, migration and invasion by targeting SPAG5 in vitro. A, Ectopic expression of miR-539 can mimic the suppression of colony formation induced by SPAG5 knockdown in PC-3 and LNCaP cells; B, Ectopic expression of miR-539 can mimic the suppression of migration activity induced by SPAG5 knockdown in PC-3 and LNCaP cells; C, Ectopic expression of miR-539 can mimic the suppression of invasion activity induced by SPAG5 knockdown in PC-3 and LNCaP cells; D, The efficiency of SPAG5 knockdown and ectopic expression of miR-539 was confirmed at protein level by western blot. (JPG 145 kb) [file 13046_2016_337_MOESM4_ESM.jpg]

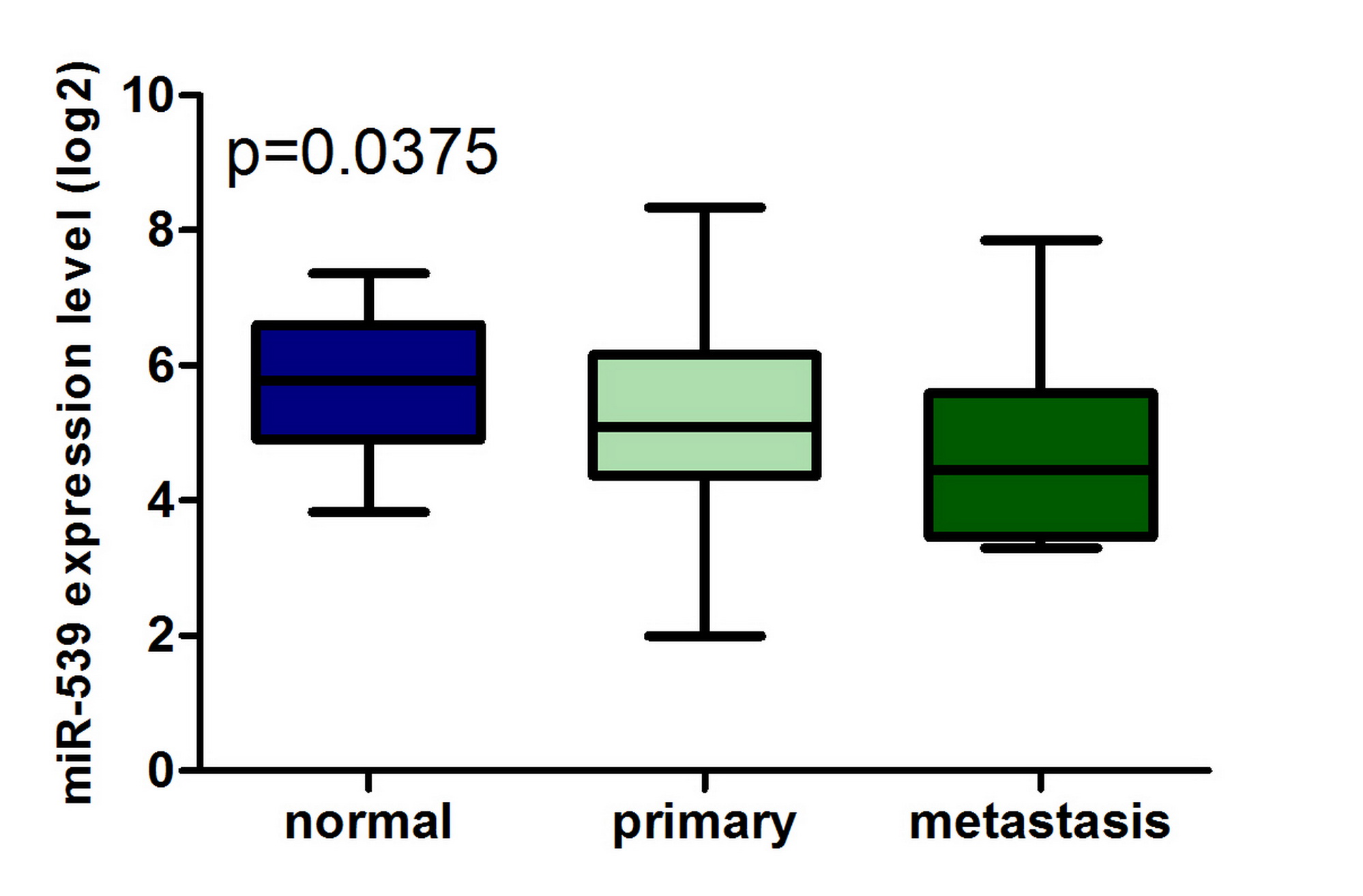

Supplement: Additional file 5: Figure S2. — miR-539 is downexpressed in primary PCa and metastatic PCa. miR-539 level was gradually decreased in normal prostate, primary PCa, and metastatic PCa samples. (JPG 173 kb) [file 13046_2016_337_MOESM5_ESM.jpg]
